# UVB Stimulates the Expression of Endothelin B Receptor in Human Melanocytes via a Sequential Activation of the p38/MSK1/CREB/MITF Pathway Which Can Be Interrupted by a French Maritime Pine Bark Extract through a Direct Inactivation of MSK1

**DOI:** 10.1371/journal.pone.0128678

**Published:** 2015-06-01

**Authors:** Hideki Tagashira, Aki Miyamoto, Sei-ichi Kitamura, Masahito Tsubata, Kazuya Yamaguchi, Kinya Takagaki, Genji Imokawa

**Affiliations:** 1 Research and Development Division, Toyo Shinyaku Co., Ltd., 7–28 Yayoigaoka, Tosu, Saga, 841–0005, Japan; 2 Research Institute for Biological Functions, Chubu University, 1200 Matsumoto, Kasugai, Aichi, 487–8501, Japan; Wenzhou Medical University, CHINA

## Abstract

Melanogenesis is the physiological process by which melanin is synthesized to protect the skin from UV damage. While paracrine interactions between keratinocytes and melanocytes are crucial for regulating epidermal pigmentation, the endothelin (EDN)-endothelin B-receptor (EDNRB) interaction is one of the key linkages. In this study, we found that a single exposure of normal human melanocytes (NHMs) with UVB stimulates the expression of EDNRB and its upstream transcription factor microphthalmia-associated transcription factor (MITF) at the transcriptional and translational levels. That stimulation can be abrogated by post-irradiation treatment with a French maritime pine bark extract (PBE). UVB stimulated the phosphorylation of p38 and c-jun N-terminal kinase (JNK), but not ERK, followed by the increased phosphorylation of MSK1 and CREB. The post-irradiation treatment with PBE did not affect the increased phosphorylation of p38 and JNK, but distinctly abrogated the phosphorylation of MSK1 and CREB. Post-irradiation treatment with the MSK1 inhibitor H89 significantly down-regulated the increased gene expression of MITF and EDNRB in UVB-exposed NHMs. Our findings indicate for the first time that the increased expression of MITF that leads to the up-regulation of melanocyte-specific proteins in UVB-exposed NHMs is mediated via activation of the p38/MSK1/CREB pathway but not the ERK/RSK/CREB pathway. The mode of action by PBE demonstrates that interrupting MSK1 activation is a new target for antioxidants including PBE which can serve as anti-pigmenting agents in a reactive oxygen species-depletion-independent manner.

## Introduction

Melanogenesis is the physiological process by which melanin is synthesized in melanocytes located in the basal layer of the epidermis to protect the skin from UV irradiation. UVB-exposed keratinocytes secrete cytokines and growth factors, including endothelin (EDN) 1 [1–5], that stimulate cellular functions, especially proliferation and melanization, of adjacent melanocytes in the epidermis. The corresponding specific receptors are constitutively expressed by human melanocytes and the binding of cytokines and growth factors to their receptors transduces intracellular signals to initiate melanogenesis through specific signaling cascades [6]. On the other hand, UVB radiation directly induces the generation of reactive oxygen species (ROS) in epidermal keratinocytes and melanocytes and stimulates stress activated protein kinases, such as p38, c-jun N-terminal kinase (JNK) or extracellular regulated protein kinase (ERK) [7]. In UVB-exposed human melanocytes, the p38 pathway predominantly contributes to the increased expression of microphthalmia-associated transcription factor (MITF) [7], a master regulator of melanocyte functions, including differentiation [8–10], proliferation [11–14], survival [15, 16] and melanogenesis [17, 18]. MITF regulates the expression of many melanogenic enzymes, melanosome structural proteins, transporters and receptors, such as tyrosinase, tyrosinase-related protein 1 (TYRP1), dopachrome tautomerase (DCT), melanosomal protein 17 (PMEL17), melanoma antigen recognized by T-cells 1 (MART1) and endothelin B-receptor (EDNRB) [19].

EDN-EDNRB binding is one of the key paracrine interactions between keratinocytes and melanocytes that regulates skin pigmentation [1, 4, 20–22]. EDN1 is a vasoconstrictor peptide originally isolated from porcine endothelial cells [23]. We first reported that human keratinocytes produce a prepro-EDN1 and then convert it by metallo-proteinases including EDN-converting enzyme α, sequentially to big-EDN1 and EDN1, which is the final secretable form [1,20,24]. UVB-exposed human keratinocytes distinctly enhance the secretion of EDN1, which triggers adjacent melanocytes in the epidermis via EDNRB to stimulate melanin synthesis [1, 20]. EDNRB is a seven-transmembrane receptor coupled with G-protein that interacts equally with all forms of EDN, EDN-1, EDN-2 and EDN-3 [25]. Mutations of those genes causes Waardenburg Syndrome Type IV, which is an auditory-pigmentary syndrome characterized by hearing loss, abnormal pigmentation of skin, hair and eyes in association with Hirschsprung disease, which is a disorder that causes blockage of the intestine [26].

The role of the EDN-EDNRB interaction was reported to induce mitogenesis and melanogenesis in melanocytes [1, 4, 20–22]. Although EDN secretion from keratinocytes stimulated by UVB has been well investigated, little is known about EDNRB expression in UVB-exposed melanocytes. EDNRB expression has been shown to increase in the epidermis when human skin is exposed to solar-stimulated radiation or UVB radiation [27–29] and in skin with lentigo senilis [30]. The finding that a dominant-negative mutant of MITF reduces the expression of EDNRB in cultured melanocytes strongly suggested that EDNRB expression is predominantly regulated by MITF [31].

**Fig 1:**
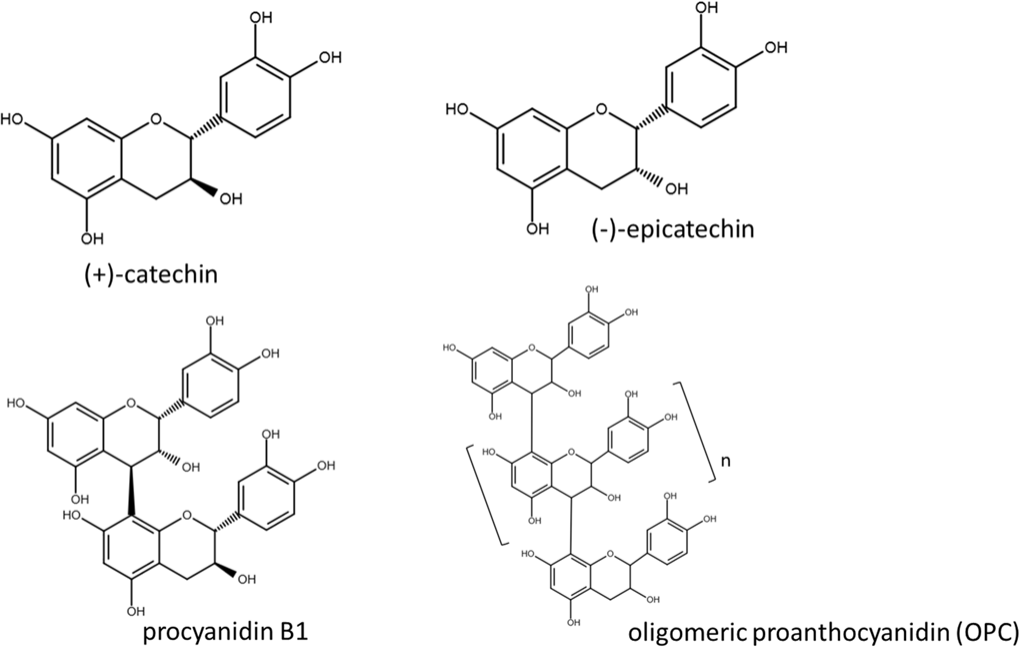
Chemical structures of major components flavonoids included in PBE.

Skin pigmentation is a major factor that prevents the skin from UV-induced damage. Pigmented skin is unwanted by people who desire a lighter skin color, and many natural products have been utilized historically for cosmetic purposes in order to obtain a lighter skin appearance [32–33]. As depicted in Fig 1, the French maritime pine (Pinus maritima) bark extract (PBE) is a complex mixture of flavonoids, which contains 72.5% polyphenol (determined by Folin Denis method) including 5% procyanidin B1, 2.98% catechin, 0.23% epicatechin and about 60% (including the percentage of dimer) oligomeric proanthocyanidin (OPC) [34–38]. PBE has been used as a traditional medicine for scurvy by maritime Indians [37–38]. PBE has potent antioxidant properties [39–41] and oral administration of PBE has protective effects on age-related diseases, such as cardiovascular dysfunction, diabetes and arthritis [38]. It was also reported that PBE by itself is highly effective in protecting the skin from UV irradiation [42]. Kim et al. demonstrated that PBE inhibits melanogenesis not via inhibition of tyrosinase but rather by inhibiting the autoxidation of melanin due to its antioxidant activity [41]. In a clinical study, oral administration of PBE at 40 or 100 mg daily for 12 weeks reduced the pigmentation of age spots [43].

Here we show that the expression of EDNRB is accentuated in UVB-exposed human melanocytes via activation of the p38/MSK1/CREB/MITF pathway where MSK1 activation is essentially responsible for CREB activation. Post-irradiation treatment with PBE does not affect p38 activation but can directly interrupt the UVB-induced activation of MSK1, which leads to abrogation of the UVB-induced up-regulation of melanocyte-specific proteins such as EDNRB. Thus, it is anticipated that PBE can serve as an anti-pigmenting agent in a ROS depletion independent manner.

## Materials and Methods

### Materials

Anti-MITF (C5), anti-EDNRB (EPR7013), anti-CREB (48H2), anti-phospho-CREB (87G3), anti-β-actin (AC-15), anti-rabbit IgG HRP-conjugated and H89 dihydrochloride were purchased from Abcam (Cambridge, MA). Anti-mouse IgG HRP-conjugated was purchased from Jackson ImmunoResearch (West Grove, PA). Antibodies for MAPK and phosphorylated MAPK, the MAPK family sampler kit and the phospho-MAPK family sampler kit were purchased from Cell Signaling Technology (Beverly, MA). Antibodies for MSK1 (C27B2) and phosphorylated (S376 and T581) MSK1 were purchased from Cell Signaling Technology. For Real-time RT-PCR, primers for β-actin (Hs_ACTB_1_SG Quantitect Primer Assay; QT00095431), EDNRB (Hs_EDNRB_1_SG Quantitect Primer Assay; QT00014343) and MITF (Hs_MITF_1_SG Quantitect Primer Assay; QT00037737) were purchased from Qiagen (Hilden, Germany). PBE (Flavangenol) which obtained by hot water extraction method from French maritime pine (*Pinus pinaster*) bark was supplied by Toyo Shinyaku (Saga, Japan).

### Melanocyte culture

Primary normal human epidermal melanocytes (NHMs) pooled from 250 individual human foreskins were purchased from Cell Systems (Kirkland, WA) and were maintained in Dermalife Ma culture medium (Lifeline Cell Technology, Walkersville, MD) supplemented with all of the supplements from the manufacturer.

### UVB source

The UVB source employed in this study was a Phillips TL20W/12RS lamp (Phillips, Eindhoven, Holland). The energy exposed was measured using a UVX radiometer with a UVX-31 sensor (UVP Inc., San Gabriel, CA).

### UVB irradiation and PBE treatment

NHMs were plated in 6-well plates at a density of 1×10^5^ cells per well in complete medium. Twenty-four h later, NHMs were washed with warmed phosphate buffered saline (PBS) once and irradiated once with 60 mJ/cm^2^ UVB in a thin layer of warmed PBS, with the lid removed. Complete medium with or without the indicated concentration of PBE was added to the well immediately after the UVB irradiation and the plates were then cultured for the indicated periods. Non-irradiated NHMs were subjected to the identical procedure but without UVB irradiation. H89 treatment was carried out instead of PBE at the indicated concentration.

### NHM viability

NHMs were plated in 96-well plates at a density of 1×10^4^ cells per well in complete medium. Twenty-four h later, the medium was removed and NHMs were washed with warmed PBS once and irradiated once with the indicated energies of UVB with the lid removed. Complete medium with or without the indicated concentration of PBE was added to the well immediately after the UVB irradiation and the plates were then cultured for 24 h. Viable NHMs were determined by a colorimetric assay with a Cell counting kit 8 (Dojin Chemical, Kumamoto, Japan), according to the manufacturer’s protocol.

### Real-time RT-PCR

Total RNAs from NHMs cultured for the indicated times were prepared using an RNeasy mini kit (Qiagen, Hilden, Germany) according to the manufacturer’s protocol. Reverse transcription and Real-time PCR reaction were used with a QuantiTect Reverse Transcription kit and a Rotor-Gene SYBR PCR kit with the gene specific primer of β-actin as a reference and the gene of interest described in Materials section according to the manufacturer’s protocol. The Real-time PCR reaction and the signal detection were carried out with Rotor-Gene Q (Qiagen, Hilden, Germany) and data analyses were carried out with Rotor-Gene Q Series Software (Qiagen, Hilden, Germany).

### Western blotting analysis

At the end of the culture, NHMs were washed twice with ice cold PBS and were lysed in RIPA buffer with the Halt Protease Inhibitor Cocktail and Halt Phosphatase Inhibitor Cocktail (Thermo Scientific, Rockford, IL). Amounts of total protein were quantitated using BCA protein reagent (Thermo Scientifc, Rockford, IL). Total proteins (5 μg/lane) were denatured by heating at 95°C in Laemmli sample buffer (BioRad, Richmond, CA) for 5 min and loaded onto 10% sodium dodecyl sulfate (SDS)-polyacrylamide gels (BioRad, Richmond, CA). After electrophoresis, proteins were transferred onto Polyvinylidene difluoride (PVDF) membranes and were immunoblotted with appropriate primary and secondary antibodies. Immunoblotted proteins were visualized using an ECL substrate (BioRad, Richmond, CA) and were detected and analyzed by ChemiDoc XR+ System and Image Lab software (BioRad, Richmond, CA).

### Statistical Analysis

All data are expressed as means ± SD (n = 3) unless noted otherwise. For pairwise comparisons, either Student’s t-test or Welch’s t-test was applied. For multiple comparisons, data were tested by one-way ANOVA, and subsequently using the Tukey or Dunnett multiple comparison test. P values less than 0.05 are considered statistically significant.

## Results

### Effect of PBE and UVB on the viability of NHMs

**Fig 2:**
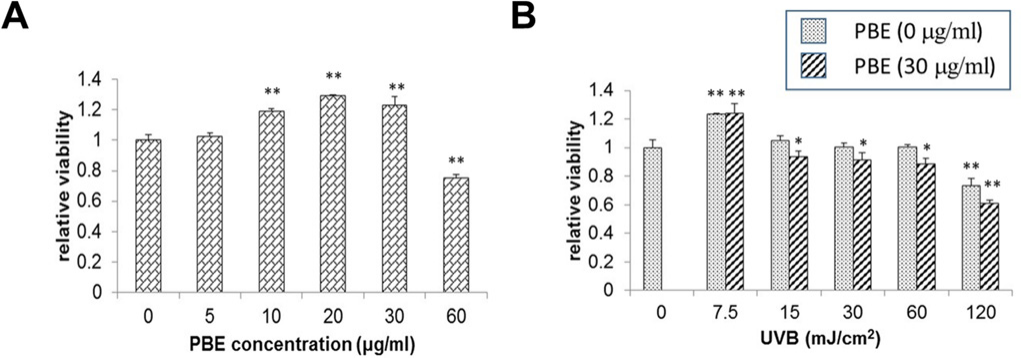
Effects of PBE treatment and UVB irradiation on NHM viability. NHMs were treated with the indicated concentration of PBE (A) and energy of UVB in the absence or presence of 30 μg/ml PBE (B). Treated NHMs were cultured for 24 h and viabilities were measured with a Cell Counting Kit-8. Error bars represent S.D. from triplicate experiments. *: P<0.05, **: P<0.01 against non-treatment with UVB or PBE.

We examined the effect of UVB irradiation and/or PBE treatment on the viability of NHMs. While treatment with PBE slightly enhanced the cell viability at concentrations of 10–30 μg/ml, it did not decrease the cell viability at concentrations less than 60 μg/ml (Fig 2A). UVB irradiation had no affect on the viability of NHMs at energy doses less than 60 mJ/cm^2^, but had a distinct effect on cell viability at a dose of 120 mJ/cm^2^ (Fig 2B). The addition of PBE to UVB-exposed NHMs at a concentration of 30 μg/ml had no substantial influence on the viability of NHMs at energy doses of less than 60 mJ/cm^2^.

### UVB stimulates the expression of EDNRB in NHMs, which is abrogated by post-irradiation treatment with PBE

**Fig 3:**
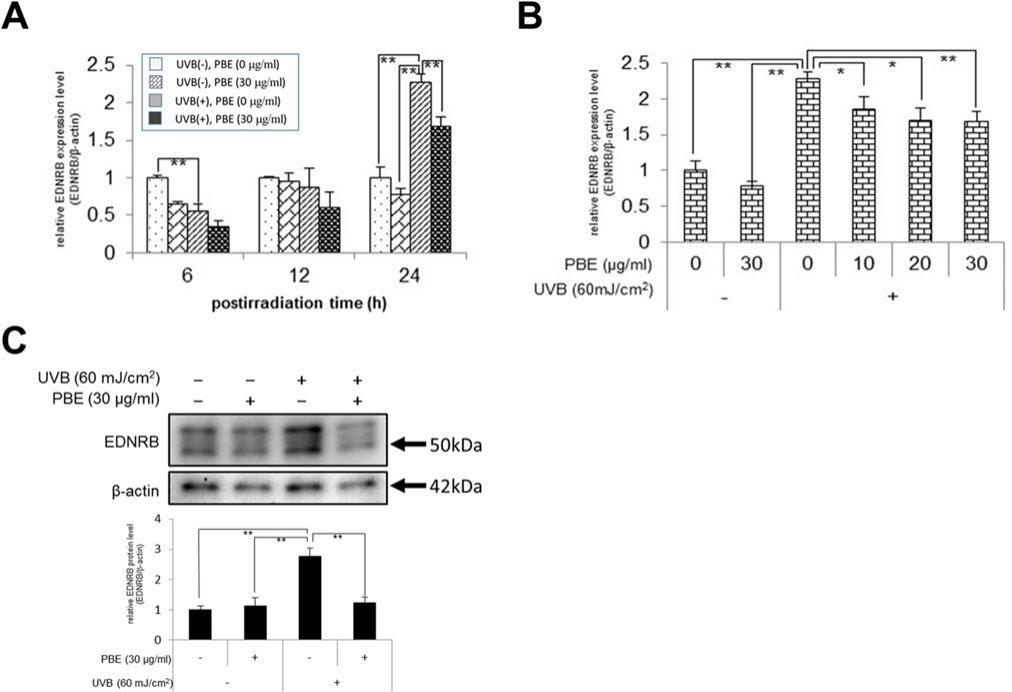
Effect of treatment with UVB and/or PBE on EDNRB expression. (A) Time course of EDNRB mRNA expression in NHMs treated without UVB in the absence of PBE, without UVB in the presence of 30 μg/ml PBE, with 60 mJ/cm^2^ UVB in the absence of PBE, or with 60 mJ/cm^2^ UVB in the presence of 30 μg/ml PBE and cultured for the indicated periods. (B) Dose dependency of PBE for EDNRB mRNA expression in NHMs at 24 h after treatment with or without 60 mJ/cm^2^ UVB in the presence of the indicated concentration of PBE. (C) Western blotting analysis for EDNRB at 24 h after treatment with or without 60 mJ/cm^2^ UVB and 30 μg/ml PBE. Protein levels were detected by specific primers and antibodies for EDNRB and β-actin as the internal control. Only the bands with 50 kDa were subjected to densitometic analysis. Error bars represent S.D. from triplicate experiments. *P<0.05 and **P<0.01 against NHMs UVB-irradiated in the absence of PBE, respectively.

We have already reported that EDNRB expression is increased by the exposure of human skin to UVB [29]. However, little was known about the biological mechanism(s) by which UVB irradiation stimulates EDNRB expression in the epidermis in vivo. Hence, we examined the effects of UVB irradiation at a dose of 60 mJ/cm^2^ on the expression of EDNRB in NHMs. Real-time RT-PCR analysis revealed that the mRNA expression level of EDNRB was significantly increased by UVB irradiation (60 mJ/cm^2^) at 24 h but not at 6 or 12 h post-irradiation (Fig 3A). When added immediately after UVB irradiation at a dose of 60 mJ/cm^2^, the enhanced expression of EDNRB mRNAs at 24 h post-irradiation was significantly abrogated by PBE at concentrations of 10, 20 and 30 μg/ml (Fig 3B). Western blotting analysis demonstrated that EDNRB protein levels were significantly increased at 24 h post-UVB irradiation (60 mJ/cm^2^), which was significantly abrogated by post-irradiation treatment with PBE at a concentration of 30 μg/ml (Fig 3C).

### UVB stimulates MITF expression in NHMs, which is abrogated by post-irradiation treatment with PBE

**Fig 4:**
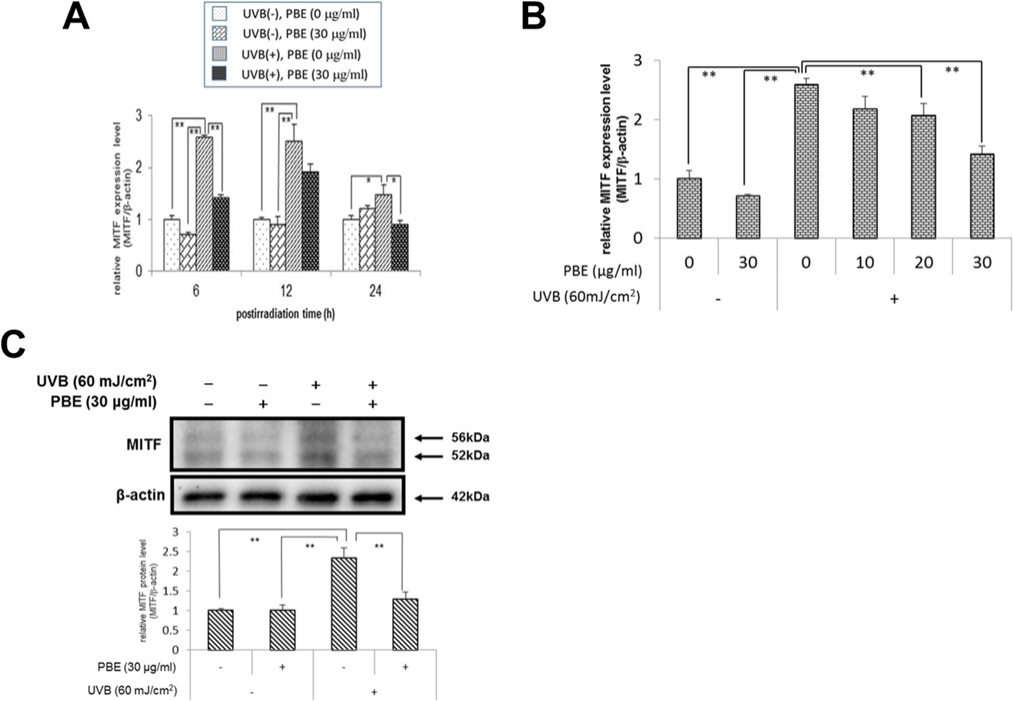
Effect of treatment with UVB and/or PBE on MITF expression. (A) Time course of MITF mRNA expression in NHMs treated without UVB in the absence of PBE, without UVB in the presence of 30 μg/ml PBE, with 60 mJ/cm^2^ UVB in the absence of PBE, or with 60 mJ/cm^2^ UVB in the presence of 30 μg/ml PBE and cultured for the indicated periods. (B) Dose dependency of PBE for MITF mRNA expression in NHMs at 6 h after treatment with or without 60 mJ/cm^2^ UVB in the presence of the indicated concentration of PBE. (C) Western blotting analysis for MITF at 12 h after treatment with or without 60 mJ/cm^2^ UVB and 30 μg/ml PBE. Expression levels were detected by specific primers and antibodies for MITF and β-actin as the internal control. Error bars represent S.D. from triplicate experiments. *P<0.05 and **P<0.01 against NHMs UVB-irradiated in the absence of PBE, respectively.

Sato-Jin et al. reported that transfection of the dominant negative form of MITF suppressed the expression of EDNRB mRNA and suggested that EDNRB gene expression occurs downstream of MITF [31]. Therefore, we examined MITF expression when NHMs were exposed to UVB irradiation and then were treated with PBE. Real-time RT-PCR analysis revealed that, when exposed to UVB at 60 mJ/cm^2^, the expression level of MITF mRNA was increased at 6, 12 and 24 h post-irradiation with a peak at 6 h post-irradiation (Fig 4A). When treated post-irradiation with PBE at a concentration of 30 μg/ml, the increased expression levels of MITF mRNA were significantly abrogated by PBE at 6 and 24 h post-irradiation. Further, the enhanced expression of MITF mRNA at 6 h post-irradiation was significantly abrogated by PBE at a concentration of 20 and 30 μg/ml in a fashion similar to those observed for the EDNRB mRNA expression (Fig 4B). Western blotting analysis demonstrated that the MITF protein level was significantly increased by UVB irradiation with 60 mJ/cm^2^ at 12 h post-irradiation, which was significantly abrogated by post-irradiation treatment with PBE at a concentration of 30 μg/ml (Fig 4C).

### CREB phosphorylation is attenuated by PBE

**Fig 5:**
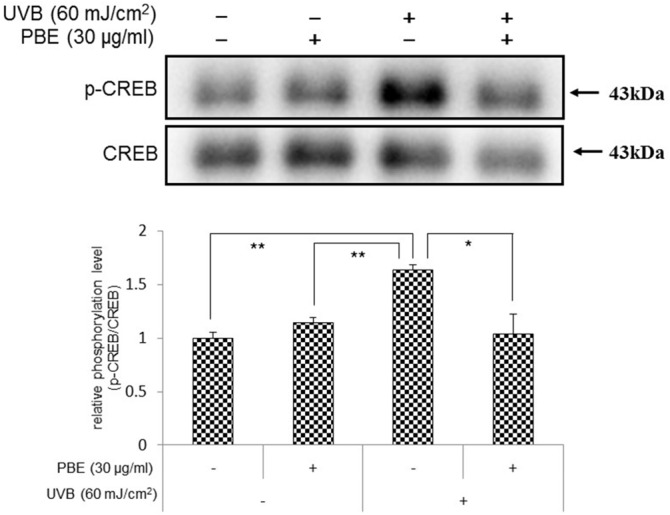
Effects of treatment with UVB and/or PBE on CREB phosphorylation. CREB phosphorylation in NHMs at 15 min after treatment with or without 60 mJ/cm^2^ UVB and/or 30 μg/ml PBE. Expression levels were detected by specific antibodies to non-phospho and phospho CREB. Error bars represent S.D. from triplicate experiments. *P<0.05 and **P<0.01 against NHMs UVB-irradiated in the absence of PBE, respectively.

Cyclic AMP response element-binding protein (CREB) is a transcription factor regulating MITF gene expression, which is activated by phosphorylation in response to various signaling molecules [30]. Since the expression of MITF and its downstream target gene EDNRB was up-regulated by UVB irradiation at the transcriptional and translational levels, and that could be abrogated by post-irradiation treatment with PBE, we next determined if the phosphorylation of CREB is increased by UVB irradiation and/or whether the post-irradiation treatment with PBE can abrogate the CREB activation. Western blotting analysis using an antibody to phosphorylated CREB revealed that the phosphorylation level of CREB was significantly increased by UVB irradiation with 60 mJ/cm^2^ at 15 min post-irradiation, which was significantly abrogated by the post-irradiation treatment with PBE at a concentration of 30 μg/ml (Fig 5). These results indicate that the up-regulation of MITF protein level is mediated via CREB activation in UVB-exposed NHMs and the post-irradiation treatment with PBE abrogates the CREB activation.

### PBE interrupts the phosphorylation of MSK1 but not ERK, JNK and p38

**Fig 6:**
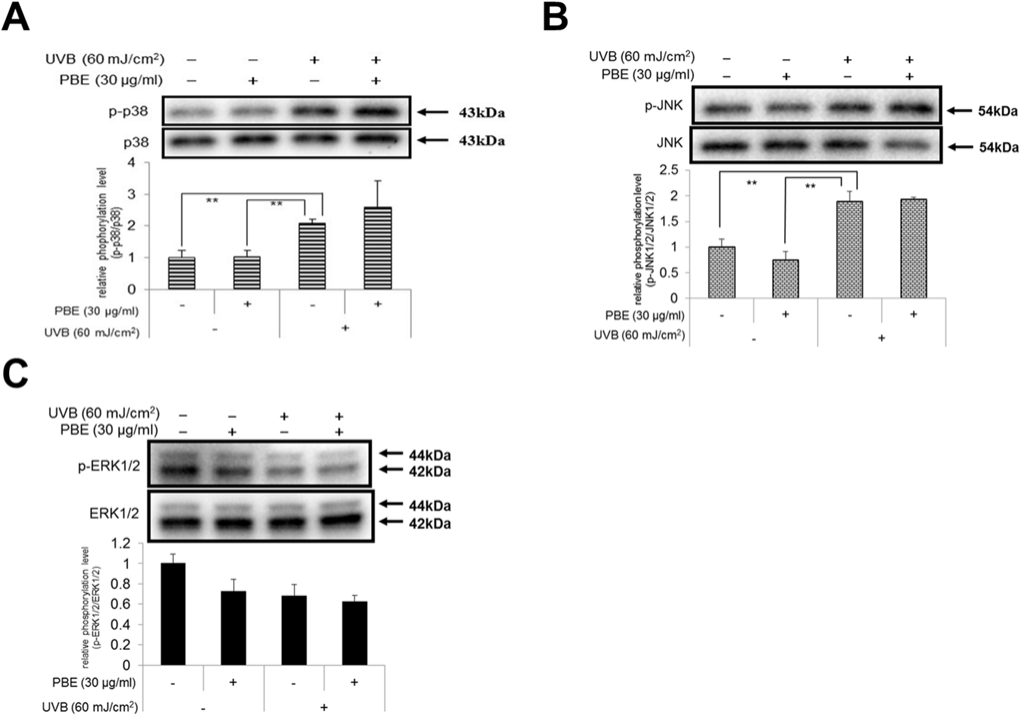
Effects of treatment with UVB and/or PBE on phosphorylation of MAPKs. The phosphorylation levels of p38 (A), JNK (B) and ERK1/2 (C) in NHMs at 15 min after treatment with or without 60 mJ/cm^2^ UVB and/or 30 μg/ml PBE. Expression levels were detected by specific antibodies to non-phospho and phospho MAPKs. Error bars represent S.D. from triplicate experiments. *P<0.05 and **P<0.01 against NHMs UVB-irradiated in the absence of PBE, respectively.

We have already reported that in UVB-exposed NHMs, the generated ROS triggers p38 and JNK but not ERK activation, leading to their downstream target CREB activation predominantly via p38 activation [7]. Based on this evidence, we next determined which signaling molecule(s) upstream of CREB are attributable to the interruption of CREB phosphorylation by post-irradiation treatment with PBE. As expected, while UVB irradiation stimulated the phosphorylation of p38 and JNK but not of ERK, the post-irradiation treatment with PBE did not abrogate the increased phosphorylation of p38 and JNK, which suggests that the interruption of CREB phosphorylation by PBE is not attributable to its effect on p38 activation (Fig 6).

**Fig 7:**
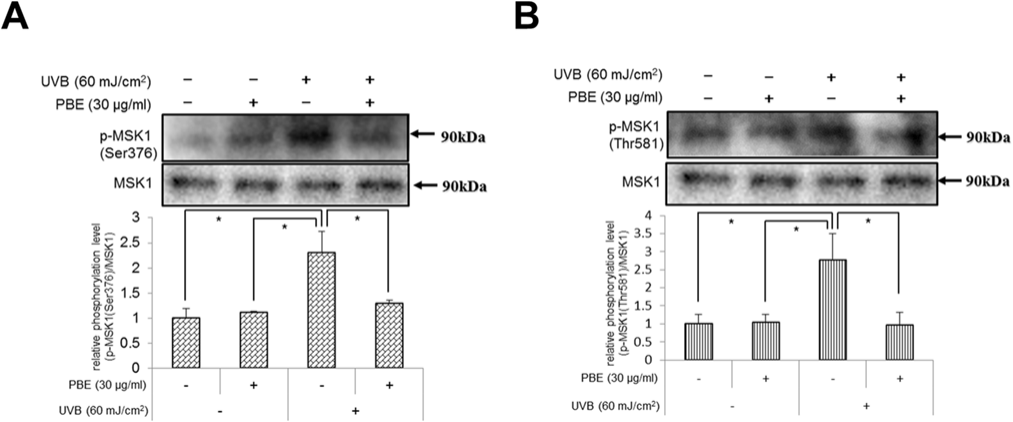
Effects of treatment with UVB and/or PBE on the phosphorylation of MSK1. The phosphorylation level of MSK1 at Ser376 residue (A) and Thr581 residue (B) at 15 min after treatment with or without 60 mJ/cm^2^ UVB and/or 30 μg/ml PBE. Expression levels were detected by specific antibodies to non-phospho and phospho MSK1. Error bars represent S.D. from triplicate experiments. *P<0.05 against NHMs UVB-irradiated in the absence of PBE.

In UVB-exposed human primary keratinocytes, the activated p38 is known to stimulate nuclear kinase mitogen-and stress activated kinase (MSK)1 which phosphorylates CREB and NFkBp65 in the nucleus during the NFkB signaling pathway [44]. Therefore, we next determined if UVB radiation stimulates the phosphorylation of MSK1 in NHMs and/or if PBE can serve as an inactivator for MSK1 even when treated post-irradiation. Western blotting analyses revealed that the phosphorylation of Ser376 (Fig 7A) and Thr581 (Fig 7B) residues of MSK1 was significantly increased 15 min following UVB irradiation, which was significantly abrogated by PBE when treated post-irradiation at 30 μg/ml (Fig 7). This suggests that the interruption of CREB phosphorylation by PBE is attributable to its abrogating effect on MSK1 activation.

**Fig 8:**
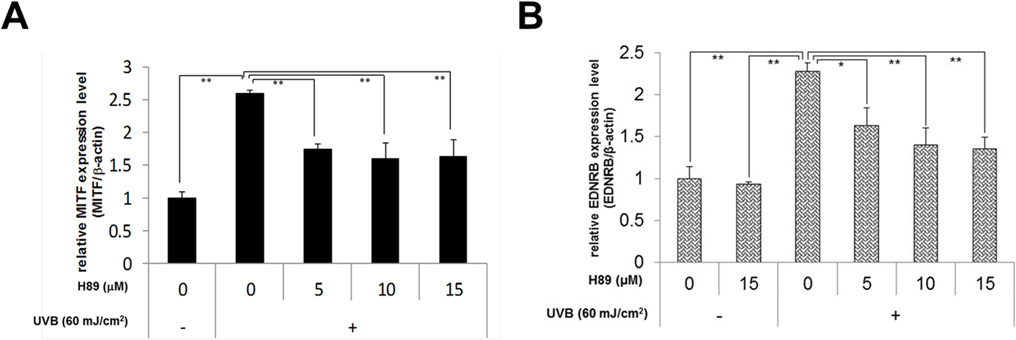
Effect of H89 on the gene expression of MITF and EDNRB in NHMs exposed to UVB. The indicated concentration of H89 was added into the medium immediately after UVB irradiation and cells were cultured for 6h (for MITF, A) or 24 h (for EDNRB, B). Total mRNAs were purified and Real-time RT-PCR was carried out with MITF or EDNRB primer and β-actin primer as the internal control. Error bars represent S.D. from triplicate experiments. *P<0.05 and **P<0.01 against NHMs UVB-irradiated in the absence of H89, respectively.

We next asked if the inhibition of MSK1 activation results in the down-regulated MITF and EDNRB expression in UVB-exposed NHMs. When the MSK1 inhibitor H89 was added to NHMs immediately after UVB irradiation, the increased expression level of MITF and EDNRB mRNA elicited by UVB irradiation was significantly abrogated by H89 (Fig 8). This suggests that the abrogation of UVB-stimulated expression of EDNRB via MITF transcription by the post-irradiation treatment with PBE is mediated via the interruption of MSK1 activation.

## Discussion

In this study, we found that a single exposure of NHMs by UVB stimulates EDNRB expression. Since the increased levels of EDNRB seem to respond to EDN1 to a greater extent than in unexposed NHMs, that finding suggests that UVB causes NHMs to become highly responsive to environmental stimuli such as EDN1 via an increased expression of the corresponding receptor, EDNRB. Consistently, we have already found that KIT ligand (KITL) up-regulates the expression of EDNRB in NHMs where the binding of ^125^I-labeled EDN1 to EDNRB increases significantly 2 days after incubation with KITL [29]. Similarly, we reported that a single exposure of NHMs with UVB stimulates expression of the KIT receptor, whose function was assessed by an increased phosphorylation following KITL stimulation [7]. Thus, it is likely that in addition to the increased production of melanogenic cytokines by UVB-exposed keratinocytes, EDNRB also plays a coordinated role in UVB-induced pigmentation by augmenting EDN1/EDNRB signaling through the accentuated function of EDNRB. In support of this, in UVB-exposed human skin where pigmentation is being stimulated, there is a significantly up-regulated expression level of EDNRB mRNA [29]. However, little is known about intracellular signaling mechanisms involved in the stimulation of EDNRB expression in UVB-exposed NHMs.

Anti-pigmentation agents have been developed as a target for various redox-sensitive biomolecules, including tyrosine hydroxylase or intracellular signaling intermediates during the melanogenesis cascade. Compounds including phytochemical agents or botanical extracts are adequate candidates for this purpose due to their distinct anti-oxidant properties [45,46]. In this study, we found that a French maritime PBE containing rich flavonoids including OPC [34–38] distinctly abrogates the increased expression of EDNRB at the transcriptional and translational levels following UVB radiation even when treated post-irradiation. PBE has a distinct antioxidant activity stronger than vitamin C and vitamin E as measured by lipid peroxidation of bovine retinal tissue [47]. Additionally, PBE possesses a potent scavenging activity for peroxynitrite (ONOO-), superoxide (•O_2_) and nitric oxide (NO•), which play a central role in inhibiting the generation of these ROS. Further, PBE up-regulates the reduced-glutathione/oxidized-glutathione ratio [41]. Owing to these strong antioxidant properties, it was anticipated that PBE has a potential to inhibit pigmentation by preventing the autoxidation of melanin [41]. Since PBE can behave as an antioxidant and a scavenger for ROS generated by UVB irradiation, its possible inhibitory effect on the increased expression of EDNRB could be accounted for by the depletion of generated ROS if treated pre-irradiation. However, our observation that post-irradiation treatment with PBE can also abrogate the increased EDNRB expression strongly suggests that PBE abrogates the up-regulation of EDNRB expression via an unknown novel signaling mechanism(s) in a ROS depletion-independent manner because the ROS lifetime is very short (e.g. lifetime of •O_2_ is 4 μs) [48], not sufficient to deplete the generated ROS when treated immediately after UVB radiation.

UVB exposure of human keratinocytes was reported to activate NFκB signaling by stimulating IKK kinase which phosphorylates IkB, causing NFκBp65 to transduce toward translocation into the nucleus during the signaling pathway downstream of the preceding p38 or JNK activation [28]. In contrast, UVB exposure of human melanocytes induces little or no activation of the NFκB pathway compared to the distinct activation of their upstream pathways such as p38 and JNK [49]. In melanocytes and melanoma, UVB has been shown to induce phosphorylation of the p38 and JNK/stress-activated protein kinase pathways, whereas NFkB remains at a constantly high expression level [50–53]. The activation of p38 or JNK following UVB radiation is mediated by initial stress-activated protein kinases, which are activated by ROS via redox-interfering mechanisms involved in protein kinases as well as their conjugated protein phosphatases [54]. Owing to these mechanisms, many antioxidants can suppress UVB-induced cellular events by scavenging generated ROS when treated pre-irradiation. This evidence indicates that the hitherto reported inhibitory effects of antioxidants on the UVB-induced activation of IKKinase leading to the diminished nuclear translocation of NFκB [55–60] may occur via the abolishing effect on the activation of p38 or JNK due to the preceding ROS depletion by pretreatment with antioxidants. Therefore, it is of considerable importance to determine the signaling mechanism(s) by which the post-irradiation treatment with PBE can abrogate the increased EDNRB expression.

We have already reported that EDNRB gene expression occurs downstream of the melanocyte-master transcription factor MITF [31]. Consistently, in this study, the gene and protein expression levels of MITF are significantly up-regulated by UVB radiation, and can be significantly abrogated by the post-irradiation treatment with PBE. This suggests that the up-regulated EDNRB expression by UVB radiation is mainly associated with the increased protein expression level of MITF and the abrogating effect of PDE on the increased expression of EDNRB is mainly attributed to the down-regulated level of MITF protein.

In NHMs, at the terminal point of the EDN1-triggered intracellular signaling cascade, the gene expression levels of melanocyte-specific proteins including EDNRB are strictly associated with the steady state levels of MITF protein. The MITF gene expression level is positively regulated by the levels of activated (phosphorylated) CREB in association with other transcription factors including SOX10, PAX3, lymphoid-enhancing factor-1 (LEF-1) and T cell factor (TCF) [61,62]. Therefore, the abrogating effect of PBE on the up-regulated expression of MITF led us to determine whether CREB phosphorylation is stimulated by UVB radiation and whether PBE abrogates this stimulation. As expected, UVB exposure of human melanocytes distinctly stimulates the phosphorylation of CREB, which is abolished by the post-irradiation treatment with PBE. This suggests that the abrogating effect of PBE on the up-regulated protein expression of MITF is mainly attributed to the interruption of CREB activation. Therefore, we next determined how the CREB is activated by UVB radiation in human melanocytes.

In our previous similar study focusing on KIT receptor expression in UVB-exposed human melanocytes, the inhibition of p38 activation by its inhibitor SB203580 results in the complete abrogation of both the up-regulated phosphorylation of CREB and the increased gene expression levels of MITF up to the non-stimulated control levels [7]. This suggests that the increased phosphorylation of CREB by UVB irradiation is mediated predominantly via the activation of p38 but not the cyclic AMP/PKA pathway. In this study, in agreement with our results and another study [7,49,63], UVB exposure of human melanocytes significantly stimulates the phosphorylation of p38 and JNK but not of ERK, whereas the increased phosphorylation of p38 and JNK is not abrogated by the post-irradiation treatment with PBE. Since p38 cannot directly phosphorylate CREB, these findings strongly suggest that the post-irradiation treatment with PBE affects signaling intermediates capable of phosphorylating CREB, which occur downstream of p38 activation. There are at least four protein kinases that have a distinct ability to phosphorylate CREB, protein kinase A (PKA), p90 ribosomal protein S6 kinase (p90RSK), MAPK-activated protein kinase-2 (MK2) and MSK1. MSK1 has a Km value much lower than the other 3 kinases, all of which are distinctly activated by p38 or ERK [64,65]. Therefore, we next determined whether MSK1 is activated by UVB radiation in human melanocytes and whether the post-irradiation treatment with PBE can abrogate the MSK1 activation.

**Fig 9:**
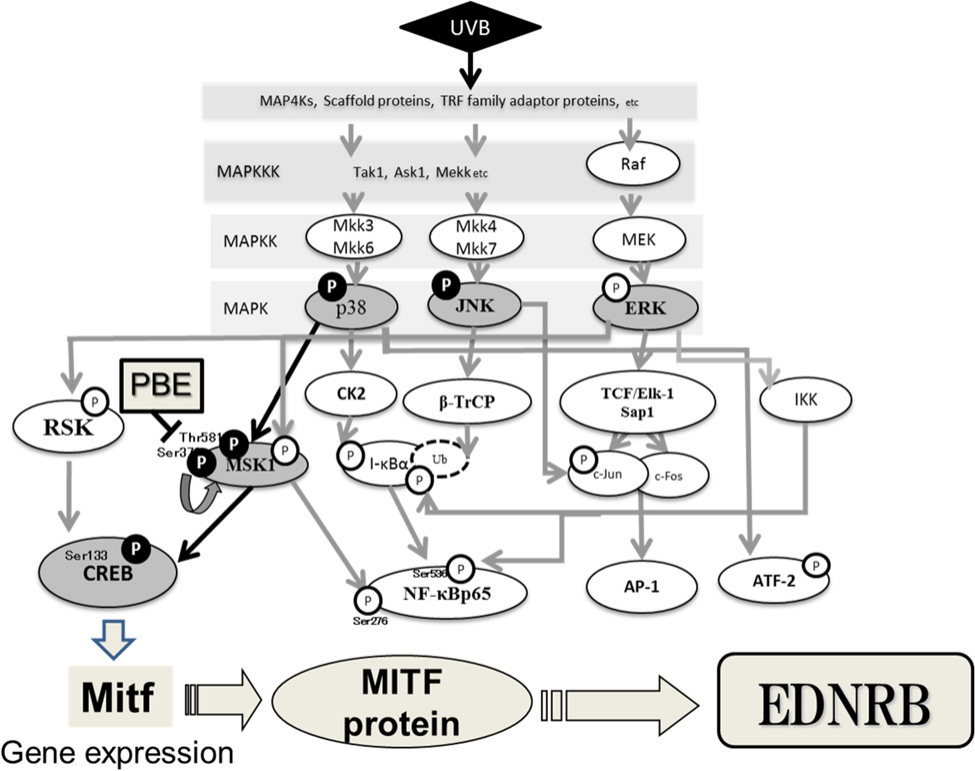
Intracellular signaling pathways leading to the UVB-induced increase in the expression of EDNRB and the site of inhibition by PBE post-irradiation treatment in human melanocytes.

MSK1 is generally expressed in epidermal keratinocytes and, as shown in Fig 9, is activated by p38 MAPK or the ERK p44/42 MAPKs through phosphorylation of either Thr581 or Ser360 [64,66,67]. The phosphorylation of Ser360 is an essential requirement for MSK1 activation [68]. Further, Ser376 is auto-phosphorylated as a result of the phosphorylation at Ser360 and Thr581 by either ERK1/2 or p38 MAPK activation [64,66,67]. However, little is known about the role of MSK1 in the signaling pathways leading to melanogenesis in human melanocytes. Western blotting analysis of MSK1 activation revealed that the phosphorylation of MSK1 at Thr581 and Ser376 was distinctly accentuated by UVB radiation, and could be significantly abrogated by the post-irradiation treatment with PBE. Since in this study ERK is not activated by UVB irradiation and PBE has no affect on ERK phosphorylation, the above findings strongly suggest that MSK1 is a signaling target of PBE, leading to the abrogation of CREB activation in UVB-exposed human melanocytes when treated post-irradiation. In this study, we also corroborated that the MSK1 inhibitor H89 significantly abrogates the increased gene expression level of MITF and EDNRB even when treated post-irradiation. This strongly indicates that MSK1 inhibition leads to the attenuated expression of MITF and EDNRB. Although the abrogated expression of MITF and EDNRB may also be attributable to the inhibition of cAMP-dependent PKA by H89, this possibility can be ruled out by the fact that the activation of CREB in UVB-exposed human melanocytes is mediated predominantly via the activation of p38 but not the cyclic AMP/PKA pathway [7], an indication that H89 treatment could not abrogate the UVB-stimulated expression of MITF and EDNRB via an interruption of the cAMP/PKA pathway. This is the first report showing that the MSK1 activation is essentially involved in the CREB activation in UVB-exposed human melanocytes and an antioxidant can directly interrupt UVB-induced MSK1 activation, which leads to the abrogation of UVB-induced up-regulation of melanocyte-specific proteins such as EDNRB.

In conclusion, as shown in Fig 9, our findings indicate for the first time that the increased expression of MITF leading to the up-regulation of the melanocyte-specific protein EDNRB in UVB-exposed human melanocytes is mediated via the activation of the p38/MSK1/CREB pathway but not of the ERK/RSK/CREB pathway. The mode of action by PBE demonstrates that the interruption of MSK1 activation is a new target for antioxidants including PBE which can serve as anti-pigmenting agents in UVB-melanosis. This study provides a deep insight into understanding of signaling mechanisms involved in UVB-accentuated expression of melanocyte-specific proteins as well as the regulatory role of redox-sensitive MSK1 in the UVB-activated melanogenic signaling pathway in human melanocytes.
